# Scrub Typhus, a Disease with Increasing Threat in Guangdong, China

**DOI:** 10.1371/journal.pone.0113968

**Published:** 2015-02-17

**Authors:** Wu De, Kou Jing, Zhang Huan, Zhou Hui Qiong, Corina Monagin, Zhong Jian Min, Huang Ping, Ke Chang Wen, Lin Jin Yan

**Affiliations:** 1 Institute of Pathogenic Microbiology, Center for Disease Control and Prevention of Guangdong, Guangzhou, People’s Republic of China; 2 Metabiota, Inc., San Francisco, California, United States of America; 3 Department of Biological Sciences, Humboldt State University, Arcata, California 95521, United States of America; University of Minnesota, UNITED STATES

## Abstract

There has been a rapid increase in the number of scrub typhus cases in Guangdong Province, China. For this reason, an epidemiologic study was conducted to understand the characteristics of scrub typhus epidemics in Guangdong. From 2006 to 2013, the incidence of human cases increased from 0.4321 to 3.5917 per 100,000 with a bimodal peak in human cases typically occurring between May and November. To detect the prevalence of *Orientia tsutsugamushi* among suspected human cases and rodents, we performed ELISA tests of IgM/IgG and nested PCR tests on 59 whole blood samples from the suspected cases and 112 spleen samples from the rodents. Suspected cases tested positive for anti-*O. tsutsugamushi* IgM and IgG 66.1% (39/59) and 50.8% (30/59) of the time, respectively. Additionally, 20.3% (12/59) of blood samples and 13.4% (15/112) of spleen samples were positive for PCR. Phylogenetic analysis revealed that there were four definable clusters among the 27 nucleotide sequences of the 56-kDa antigen genes: 44.4% Karp (12/27), 25.9% Kato (7/27), 22.2% Gilliam (6/27) and 7.4% TA763 (2/27). We concluded many suspected cases may result in diagnostic errors; therefore, it is necessary to perform laboratory tests on suspected cases in hospitals. The high infection rate of *O. tsutsugamushi* among the limited rodents tested suggested that further rodent sampling throughout the province is necessary to further define high-risk areas. Furthermore, the multiple co-circulating genotypes of *O. tsutsugamushi* play a key role in the pervasiveness of scrub typhus in the Guangdong area.

## Background

Approximately one billion people are affected with Scrub typhus [1]. Scrub typhus caused by *O. tsutsugamushi* is a major cause of acute febrile illnesses in the Asia-Pacific region. Descriptions of scrub typhus have been found in Chinese writings as early as 313 A.D. and were first described in modern literature in Japan in 1810 [2,3]. Moreover, the *O. tsutsugamushi* strain Ikeda was originally isolated from a patient in 1979 in the Niigata Prefecture, Japan [4]. Scrub typhus is an endemic disease threatening a wide area of the Asia-Pacific rim, extending from Afghanistan to China, Korea, the islands of the southwestern Pacific, and northern Australia [5].

Mainland China initially considered Scrub typhus an infectious disease in 1952. Nationwide surveillance data showed that approximately 315-1,844 cases had been reported annually before 1985 [6]. However, this disease was not reported in northern China (north of the Yangtze River) until 1986 when it appeared in the Shandong Province [7] and has since rapidly expanded further north. Subsequently, over 2,000 cases were reported on annually [8]. Since scrub typhus is not a notifiable infectious disease in mainland China, the registration of scrub typhus cases was aborted in 1990. As a result this has led to an underestimation of its prevalence and risk. Due to an increasing number of cases and multiple outbreaks in the past two decades [8,9,10], scrub typhus surveillance was restarted by the China Information System for Disease Control and Prevention (CISDCP) in some Chinese provinces beginning in January 2006.

Since 1948 when the first case of scrub typhus was reported in the subtropical zone of southern China in the Guangdong Province, it has been considered the main focus of scrub typhus studies [11]. However, the local epidemiological characteristics of scrub typhus and the molecular genotype of *O. tsutsugamushi* remain unclear. For this reason, this study analyzed the surveillance data from 2006 to 2013 in order to describe the epidemiological characteristics of scrub typhus. We collected specimens from suspected human patients and rodents to perform antibody tests and genetic typing based on the 56-kDa type-specific antigen (TSA).

## Materials and Methods

### Ethics statement

This study was carried out in strict accordance with Animal Protection Law of the People’s Republic of China and with the local governments’ approvals and permits to conduct this study. The rodents in this study were not an endangered or protected species and were collected from hills and villages in Qingyuan and Shantou area. The protocol was approved by Guangdong Provincial Animal Care and Use Committee (Permit Number: 140025) and Medical Ethical Committee of the Center for Disease Control and Prevention of Guangdong Province. All surgery was performed under diethyl ether anesthesia and all efforts were made to minimize suffering. All participants were voluntary with written informed consents and the data was analyzed anonymously.

### Case definition

The definition for a suspected case was a fever, a field exposure history within 3 weeks before onset, and either lymphadenectasis or rash. Additionally, suspected cases could simultaneously present with a fever, lymphadenectasis, and a rash. The criterion for a clinical case was the definition of a suspected case plus an eschar/ulcer, or the concurrent presentation of a fever and an eschar/ulcer along with a history of previous epidemic contact. Confirmed cases were suspected cases or clinical cases with one of the following laboratory test results: Weil–Felix OX_K_ agglutination titer ≥1:160, a 4-fold increase in immunoglobulin G (IgG) using indirect immunofluorescent assay (IFA), a positive result in pathogenic isolation, and/or a positive result with nested polymerase chain reaction (PCR). Following these definitions, scrub typhus cases diagnosed by physicians in Guangdong were reported to the local CDC through the CISDCP.

### Data sources

Surveillance data, including demographic information, location of onset, and date of reported cases of scrub typhus from 2006 to 2013, was obtained through the CISDCP in order to describe the epidemiological characteristics of scrub typhus.

### Specimen collection

**Fig 1:**
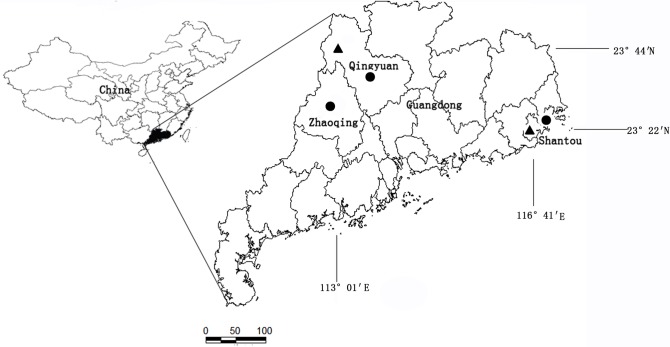
Map of the geographic areas in Guangdong showing human cases and rodent study sites, “▲”signs indicates rodent study sites, “●”signs indicates human case study sites.

Blood specimens from suspected patients of scrub typhus were collected from Qingyuan, Zhaoqing and Shantou cities from 2012 to 2014 [Fig. 1]. In total, 59 specimens were collected from suspected patients with a fever (>38°C) of unknown origin (FUO) occasionally accompanied by headache, myalgia, nausea, vomiting, diarrhea, cough, and/or breathlessness. These patients were primarily farmers who lived in rural areas. Whole blood samples were aliquoted into two tubes, one dry tube and one with Ethylenediaminetetraacetic acid (EDTA). The tubes were shipped at 4°C to the Guangdong Provincial Center for Disease Control and Prevention (GDCDC) for further analysis.

A total of 112 rodents were captured by randomly setting live traps in fields and densely populated areas [12] from 2012 to 2013 in Qingyuan and Shantou districts [Fig. 1]. All rodents were anesthetized while spleen samples were dissected and collected into cryotubes. Then the samples were then transported to GDCDC and stored in −80°C for further detection of *O. tsutsugamushi*. The rodent species sampled in this study were diverse, including 12 *Bandicota indica*, 20 *Rattus norvegicus*, 51 *Rattus loseae*, 12 *Rattus flavipectus*, and 17 *Suncus murinus*.

### Sample preparation and DNA extraction

Anti-coagulated and coagulated blood samples were centrifuged for 10 min at 3,000 g, then the buffy coats and sera were removed and stored at −20°C for *O. tsutsugamushi* nucleotide and antibody tests. Spleen samples from rodents were homogenized using preCellys 24 (Bertin Technologies, France). The homogenate was centrifuged for 10 min at 5,000 g, and the supernatant was removed for DNA extraction. Genomic DNA was extracted using DNeasy tissue kits (QIAGEN, Hilden, Germany) according to the manufacturer’s protocol.

### PCR amplification

Nested PCR amplification reactions were performed using HotStarTaq Master Mix Kit (QIAGEN, Hilden, German). The reaction consisted of 2X Reaction Mix (a buffer containing 0.2mM of each dNTP, 1.5 mM MgCl_2_ and 2.5 units HotStarTaq DNA Polymerase) and 0.4 μM specific primers for 56-kDa type specific antigen (TSA) gene. The forward and reverse primers were designed previously [13]. Ten μl of extracted DNA and 1 μl of the first PCR products were used respectively as template DNA in the first and the second PCRs. The two reactions were added respectively to a final volume of 25 μl. The cycling conditions for the nested PCRs were: an initial cycle at 95°C for 15 min, followed by 40 cycles at 94°C for 30 s, 57°C for 40 s and 72°C for 1 min; and finishing with a final incubation at 72°C for 10 min. The band of PCR amplicons visualized after electrophoresis were subsequently excised from 1% agarose gel and purified by use of a QIAGEN gel extraction kit (QIAGEN, Hilden, Germany).

### Nucleotide sequences and phylogenetic analysis

The purified PCR products were sequenced with an ABI 3100 Genetic Analyzer (Applied Biosystems, USA) with Life Technologies Company. The resulting sequences were compared to those available in nucleic acid databases using the BLASTN database (National Center for Biotechnology Information) in order to determine the closest relatives. The nucleotide sequences were analyzed with MEGA software version 5.0. Phylogenetic trees were constructed by a neighbor-joining method after 1000 bootstrapped replicates [14].

### ELISA

Forty two human blood samples were tested for *O. tsutsugamushi* infection using Scrub Typhus Detect IgG /IgM ELISA System (InBios international inc, Seattle, USA) according to the manufacturer’s protocol. OT-derived recombinant antigens were briefly coated in each polystyrene microtiter well. For each sample, 4 μl of undiluted serum was diluted to a final concentration of 1:100 using a sample dilution buffer for scrub typhus IgG/IgM. The results were considered positive when the optical density (OD) units of IgM/IgG were ≥0.3/0.5.

### Nucleotide sequence accession numbers

The partial 56-kDa TSA gene sequences from this study are available in GenBank with the following accession numbers: KJ188179-KJ188198 and KM492919-KM492925.

## Results

### Intensity of the epidemic

**Fig 2:**
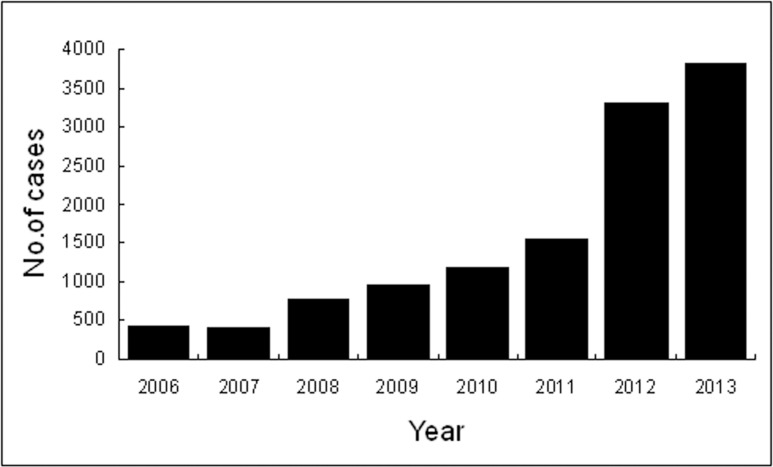
Number of reported annual cases of scrub typhus in Guangdong, China, from 2006 to 2013.

A total of 12,325 cases were reported in Guangdong Province from 2006 to 2013, including 11,026 clinical cases, 713 confirmed cases, 586 suspected cases, and 17 deaths. Overall, the number of reported cases has increased yearly during surveillance periods and reached its highest peak in 2013 (Fig. 2). These reported cases were from 21 cities; of which, Guangzhou City reported the highest number of scrub typhus cases (34.3%) during the surveillance period, followed by Zhaoqing (17%), Yunfo (6.66%) and Qingyuan (5.67%). A noticeable increase in the annual incidence was observed from 0.4321 to 3.5917 per 100,000 people in Guangdong province from 2006 to 2013. The highest incidence of the disease (17.9 /100,000) was recorded in Zhaoqing City in 2013, followed by Yunfo (9.26/100,000), Qingyuan (8.58/100,000), and Shaoguan (7.56/100,000).

### Temporal distribution

**Fig 3:**
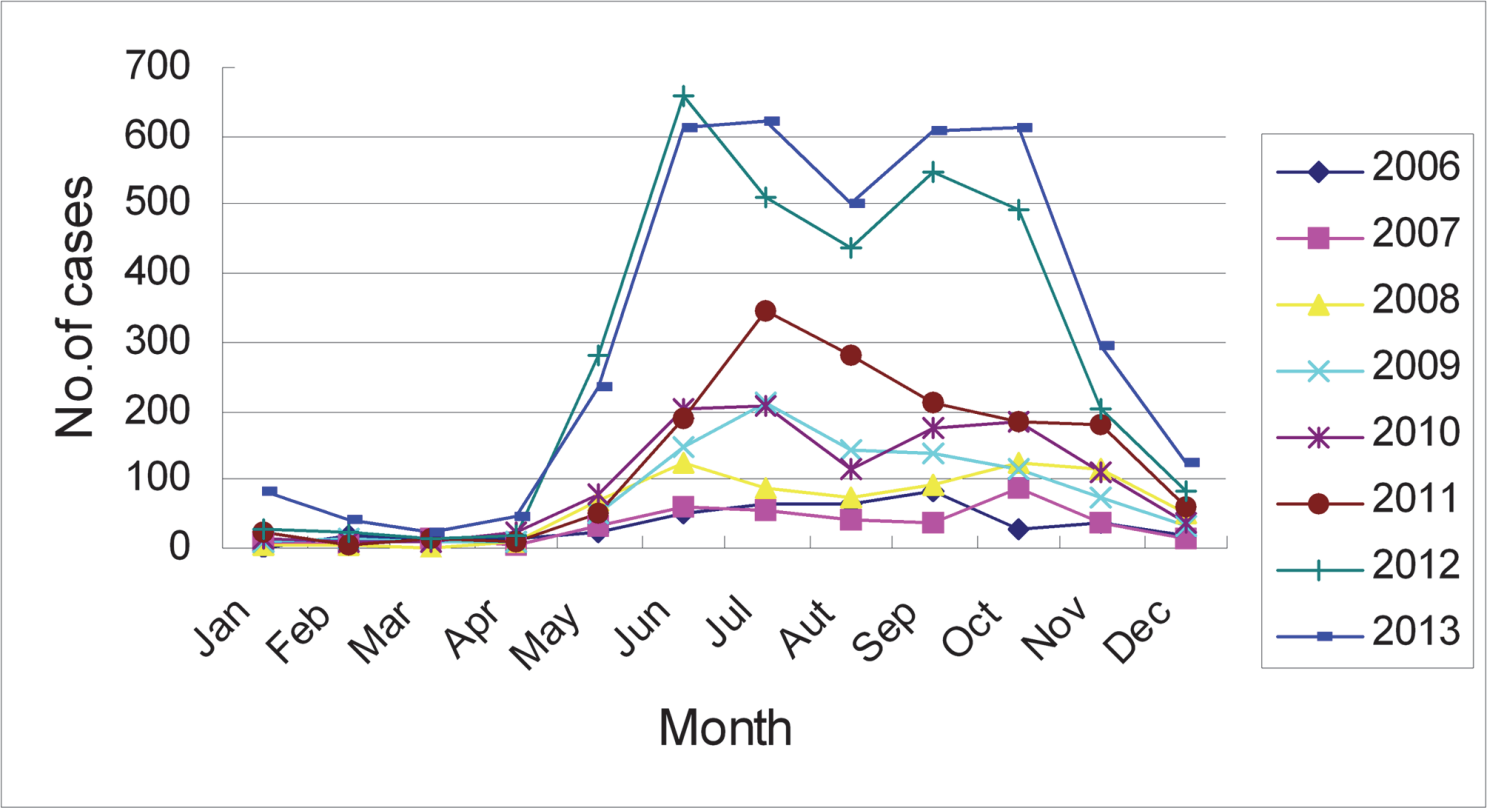
Monthly distribution of reported cases of scrub typhus in Guangdong, China, from 2006 to 2013.

The cases occurred predominantly during May to November each year and the highest prevalence was observed in 2013. The epidemiological curve of scrub typhus based on the GDCDC surveillance network data are shown in Fig. 3. All curves depicting the number of cases rapidly increased from May and reached their bimodal peak between June and October from 2006 to 2013 with the maximum number of cases during the periods of June-July and September-October. Interestingly, this characteristic was not observed in 2009 and 2010. The number of reported cases began to quickly decrease from November and baselined in December every year.

### Other epidemiological features

The distribution of cases by age clearly showed a higher incidence of scrub typhus in people older than 40 years of age (75.7%). When patients were categorized by occupation, there were a significantly higher number of infections among farmers (59.9%) than among those in the other eighteen job groups. A total of 5,762 male and 6,563 female cases were reported to GDCDC through the surveillance network with the overall male-to-female ratio of 0.878:1 from 2006 to 2013.

### Nested PCR and ELISA

Nested PCR was performed on 59 specimens collected from the suspected cases and 112 spleens from rodents for the detection of 56 kDa-TSA gene. Nested PCR assays tested positive for *O. tsutsugamushi* in 12 out of 59 (20%) specimens from suspected cases and in 15 out of 112 (13%) specimens from rodents. Of the 15 positive rodent specimens distributed amongst three species, 8/51, 4/20 and 3/12 were from *R. losea, R. norvegicus* and *R. flavipectus*, respectively (Table 1).

**Table 1:** Strains of *O. tsutsugamushi* collected in Guangdong in this study.

**Strain**	**Collection date**	**Source**	**Geographical origin**	**Sample**	**GenBank accession no**	**Cluster**
China/GDqy97	2012.8	*R. losea*	Qingyuan	Spleen	KJ188189	Kato
China/GDqy99	2012.8	*R. losea*	Qingyuan	Spleen	KJ188187	Kato
China/GDqy160	2012.8	*R. losea*	Qingyuan	Spleen	KJ188196	Gilliam
China/GDqy107	2012.8	*R. losea*	Qingyuan	Spleen	KJ188193	Gilliam
China/GDqy108	2012.8	*R. losea*	Qingyuan	Spleen	KJ188194	Gilliam
China/GDqy111	2012.8	*R. losea*	Qingyuan	Spleen	KJ188191	Kato
China/GDqy121	2012.8	*R. losea*	Qingyuan	Spleen	KJ188188	Kato
China/GDqy137	2012.8	*R. losea*	Qingyuan	Spleen	KJ188195	Gilliam
China/GDst13F	2011.6	*R. norvegicus*	Shantou	Spleen	KJ188179	Karp
China/GDst10F	2011.6	*R. flavipectus*	Shantou	Spleen	KJ188183	Karp
China/GDst37P	2011.6	*R. norvegicus*	Shantou	Spleen	KJ188184	Karp
China/GDst33G	2011.6	*R. norvegicus*	Shantou	Spleen	KJ188186	TA763
China/GDst35G	2011.6	*R. norvegicus*	Shantou	Spleen	KJ188192	Gilliam
China/GDys19G	2012.6	*R. flavipectus*	Qingyuan	Spleen	KJ188180	Karp
China/GDys21G	2012.6	*R. flavipectus*	Qingyuan	Spleen	KJ188181	Karp
China/GDfk13017	2013.6	*Homo sapiens*	Zhaoqing	Blood	KJ188185	Karp
China/GDQY13052	2013.8	*Homo sapiens*	Qingyuan	Blood	KJ188197	Gilliam
China/GDQY13053	2013.8	*Homo sapiens*	Qingyuan	Blood	KJ188198	TA763
China/GDyj13016	2012.6	*Homo sapiens*	Yangjiang	Blood	KJ188190	Karp
China/GDst103	2012.1	*Homo sapiens*	Shantou	Blood	KJ188182	Karp
China/GD-E14079	2014.5	*Homo sapiens*	Zhaoqing	Blood	KM492919	Kato
China/GD-E14084	2014.6	*Homo sapiens*	Zhaoqing	Blood	KM492920	Karp
China/GD-E14102	2014.6	*Homo sapiens*	Zhaoqing	Blood	KM492921	Karp
China/GD-E14110	2014.6	*Homo sapiens*	Zhaoqing	Blood	KM492922	Karp
China/GD-E14080	2014.5	*Homo sapiens*	Zhaoqing	Blood	KM492923	Karp
China/GD-E14091	2014.6	*Homo sapiens*	Zhaoqing	Blood	KM492924	Kato
China/GD-E14088	2014.6	*Homo sapiens*	Zhaoqing	Blood	KM492925	Kato

Scrub typhus serology testing was conducted for the detection of specific immunoglobulin (Ig) G and M using IgM and IgG ELISA with OT-derived recombinant antigens. Results indicated that 39 samples were positive for anti-*O. tsutsugamushi* IgM, 30 samples were positive for anti-*O. tsutsugamushi* IgG, and 28 samples were positive for both IgG and IgM.

### Molecular typing and phylogenetic analysis

**Fig 4:**
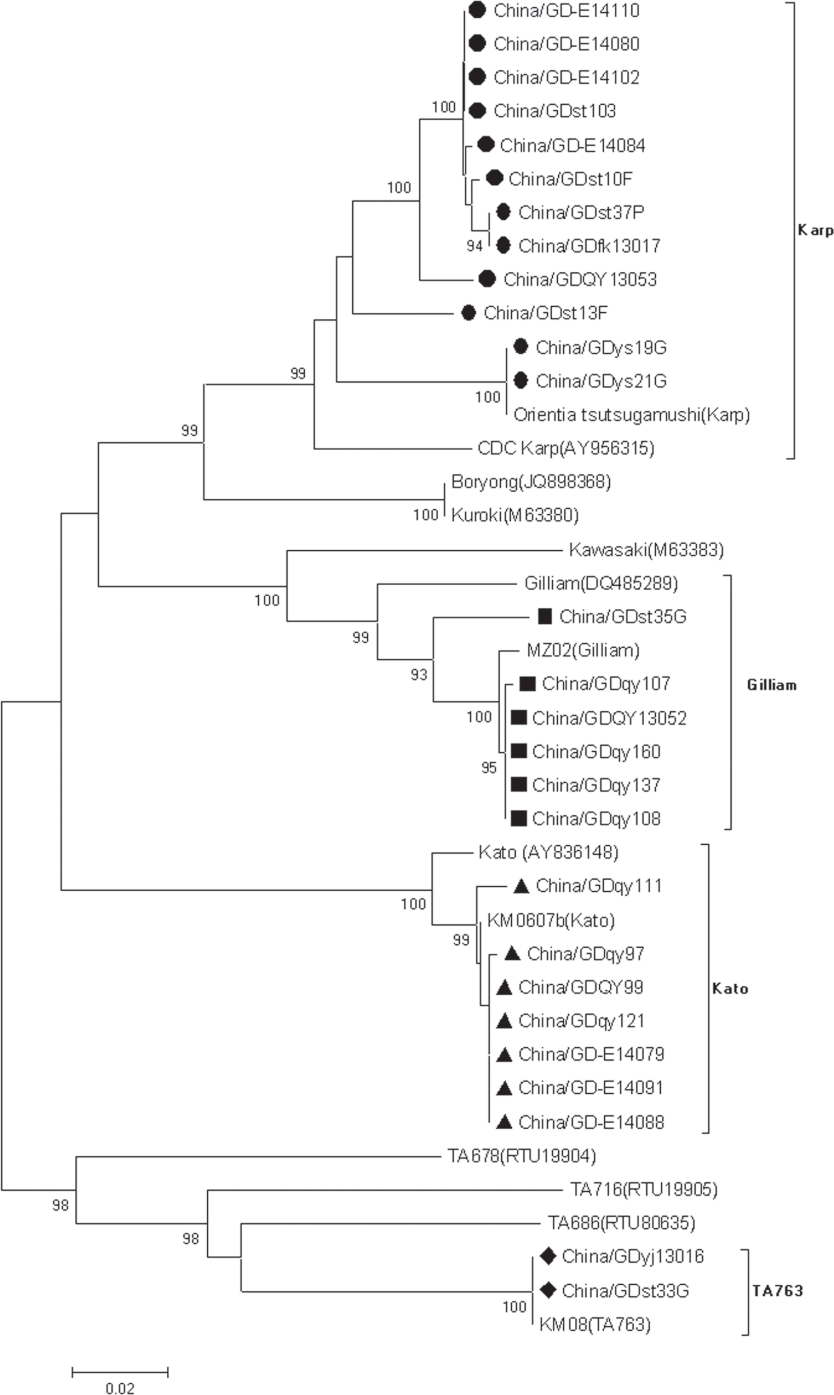
Phylogenetic tree based on the 56-kDa TSA partial gene sequence of 37 strains of *O. tsutsugamushi*. The tree was constructed by the neighbor-joining method. Bootstrap support values > 75 are shown. The 27 representative strains described in this study are designated in different shape black-markers. *O. tsutsugamushi* strains were identified using the nomenclature of strain name (GenBank accession no).

Fig. 4 shows the phylogenetic analysis of the 56 kDa-TSA gene from 37 strains including 27 representative isolates described in this study and 10 global reference strains available from GenBank. All 27 sequences in this study were divided into four distinct clusters: Karp, Kato, Gilliam and TA763. Most of the contemporary Guangdong strains (44.4%, 12/27) that displayed nucleotide sequence identity in the range of 91.9–93.0% with the Karp reference strain (AY956315), clustered with the Karp-type. Six strains (22.2%, 6/27) that displayed nucleotide sequence identity in the range of 93.2–93.7%, demonstrated the highest similarity to the Gilliam reference strain (DQ485289). Four strains (25.9%, 7/27) that showed 97.5–97.99% nucleotide identity with the Kato reference strain (AY836148) were most closely related to the Kato-type. Two strains (7.4%, 2/27) that had a percent nucleotide identity to the TA763 strain ranging from 99.68% to 100% were closely related to the TA763 prototype strain isolates (KM08).

## Discussion

Scrub typhus was known to be endemic and present in mainland China for over 2,000 years. The pathogen, *O. tsutsugamushi*, has been circulating among chigger mites, rodents, and humans, which has resulted in the rapid expansion of scrub typhus [15]. Scrub typhus was primarily endemic south of the Yangtze River, however, the endemic areas shifted after 1986 with novel affected areas of scrub typhus successively found in areas north of the Yangtze River. Currently, there are at least 28 out of 34 provinces and 574 counties that are affected by scrub typhus infections [16]. The epidemic areas extend from eastern Taiwan and Fujian to western Yunnan, and from southern Hainan, Guangdong and Guangxi to northern Inner Mongolia. The epidemic areas also include Sinkiang, Sichuan, south of Tibet, and three provinces in northeastern China. As a result of natural foci expansion, a rapid increase in case reports occurred during 2006–2013 with a total of 27,391 confirmed cases reported [11]. During the past seven years, 29.6% (8,120/27,391) of these cases were reported in Guangdong with a dramatic increase in cases being observed in the past eight years. The number of scrub typhus cases peaked in 2013 with 10.87 times more reported cases than in 2006. According to physicians in surveillance hospitals, most of them attribute this dramatic rise to increased physician awareness and infection rates of *O. tsutsugamushi*.

Scrub typhus is divided into four types according to temporal distribution in China; summer type, autumn type, autumn-winter type and whole-year type [15]. In previous studies, the summer type mainly prevailed in the southeastern coastal provinces including Fujian and Zhejiang (between the northern latitude 25°–31°) from May to October and peaked in June-July. The autumn type and autumn-winter type mainly prevailed between northern latitude 31°–40°, such as Jiangsu and Anhui, from September to December and peaked in October-November. The whole-year type mainly prevailed in Guangdong (below 25° North latitude) [14,17]. In this study, we reported that scrub typhus occurred throughout the entire year, but prevailed from May to November. We observed a previously undescribed result in which two peaks were observed in the summer and autumn in most years. In mainland China, the summer scrub typhus was predominantly caused by the Gilliam type, whereas the autumn scrub typhus was typically caused by the Kawasaki type [18]. Since *O. tsutsugamushi* has been reported to have a variety of serotypes in the same epidemic area [12,19], we infer that the two peaks could potentially be caused by different dominant genotypes.

While the majority of cases (94.3%) were not definitively diagnosed by laboratory tests, 5.7% of cases were confirmed by laboratory tests according to surveillance data. Since there are no specific laboratory diagnostic tests for scrub typhus in hospitals, many cases are diagnosed by patients’ clinical manifestation. Being that the presence of an eschar in a patient with acute febrile illness is of very high diagnostic value for scrub typhus infection, the majority of doctors diagnose it based on the presence of an eschar. Unfortunately, not all scrub typhus patients present with an eschar [20,21]. Therefore, to verify the infection rate of *O. tsutsugamushi* among patients without an eschar, we collected blood samples from the suspected patients to perform antibody and nucleotide tests for it. In general, indirect immunofluorescent assay (IFA), which is a diagnostic method for scrub typhus, is sensitive and specific in detecting the antibody to *O. tsutsugamushi*. However, several studies reported that the sensitivity and specificity of the recombinant antigen assays were equivalent to an IFA [22,23]. In this study, we performed antibody tests using ELISA with a recombinant protein as the antigen. The results showed that 73.8% and 11.9% of samples were positive for antibody and nucleotide tests for *O. tsutsugamushi,* respectively. It can be inferred that many cases without an eschar may result in diagnostic errors. For this reason, we strongly suggest that it is necessary to perform laboratory tests on suspected cases in hospitals.

Rodent species are the main hosts for the chigger mites of the genus *Leptotrombidium* and are widely distributed in urban and rural areas. *R. losea* and *R. norvegicus* are dominant species in Guangdong [24] and are considered to be the most significant factor of scrub typhus’s pervasiveness due to the high infection rate of *O. tsutsugamushi.* To illustrate this we collected spleen samples from wild rodents to evaluate the prevalence of *O. tsutsugamushi*. The average occurrence of *O. tsutsugamushi* among these rodents was 13.4%, which was much higher than the result reported in Korea (4.5–12.4%) [25]. More specifically, *R. losea* and *R. norvegicus* tested positive 15.7% and 20% of the time, respectively. This indicated a high risk of scrub typhus infections in the natural environment, which results in a high rate of occurrence in Guangdong.

Multiple serotypes of *O. tsutsugamushi* have been identified in China, including Gilliam, Kato, Karp, Kuroki, Shimokoshi, TA763, and Kawasaki strains. The Karp, Kato and TA763 serotypes are prevalent in Taiwan [26] and the Gilliam serotype is mainly in southern China. The aim of this study was to clarify the previously unknown genotypes of *O. tsutsugamushi* in Guangdong Province. In addition, this study is also unique since it was the first to genetically characterize the strains of *O. tsutsugamushi* from patients and rodents with scrub typhus infections in Guangdong. We amplified a 672 bp region containing VDI, VDII and VDIII of the *O. tsutsugamushi*-specific 56-kDa genes by nested PCR. Twenty samples were amplified with PCR and their sequences were analyzed. The Karp genotype was the most common at a rate of 40%, which is consistent with the rates in Cambodia (43.5%) and Taiwan (37%), but differs with the rate in Korea (the Boryong 76.6%) [25,26,27]. China/GDst33G and China/GDyj13016 strains showed 99.7%-100% similarity to the KM08 strain (GU120153), which was identified as TA763 in Taiwan [26] and the first genotype reported in Guangdong.

In conclusion, the results of this study confirmed that scrub typhus is an important public health concern in Guangdong. We suggest the high infection rate of *O. tsutsugamushi* among rodents may result in a high risk of scrub typhus infections. Since laboratory diagnostics are commonly lacking for the detection of the disease in Guangdong, it is necessary to improve diagnostic methods in order to confirm cases of scrub typhus in hospitals. We confirmed the Karp genotype is most common in Guangdong province and identified the presence of the TA763 genotype, which had not previously been reported in Guangdong. The results of this study confirm the presence of various genotypes including Karp, Gilliam, Kato and TA763 in this area.
